# The need for Pan‐European automatic pollen and fungal spore monitoring: A stakeholder workshop position paper

**DOI:** 10.1002/clt2.12015

**Published:** 2021-05-02

**Authors:** Fiona Tummon, Lucas Alados Arboledas, Maira Bonini, Benjamin Guinot, Martin Hicke, Christophe Jacob, Vladimir Kendrovski, William McCairns, Eric Petermann, Vincent‐Henri Peuch, Oliver Pfaar, Michaël Sicard, Branko Sikoparija, Bernard Clot

**Affiliations:** ^1^ Federal Office of Meteorology and Climatology MeteoSwiss Payerne Switzerland; ^2^ Andalusian Institute for Earth System Research (IISTA‐CEAMA) Granada Spain; ^3^ Agency for Health Protection of Metropolitan Area of Milan (ATS) Milan Italy; ^4^ Laboratoire d'Aérologie CNRS UPS—Université Toulouse III Toulouse France; ^5^ Réseau National de Surveillance Aérobiologique Brussieu France; ^6^ Bavarian State Ministry of Health and Care Munich Germany; ^7^ EUMETRep Brussels Belgium; ^8^ WHO European Centre for Environment and Health Bonn Germany; ^9^ ECOMET Brussels Belgium; ^10^ EIG EUMETNET Secretariat Brussels Belgium; ^11^ Copernicus Atmospheric Monitoring Services European Centre for Medium‐Range Weather Forecasts Reading UK; ^12^ Department of Otorhinolaryngology, Head and Neck Surgery Section of Rhinology and Allergy University Hospital Marburg Philipps‐Universität Marburg Marburg Germany; ^13^ CommSensLab Department of Signal Theory and Communications Universitat Politècnica de Catalunya Barcelona Spain; ^14^ Ciències i Tecnologies de l'Espai‐Centre de Recerca de l'Aeronàutica i de l'Espai/Institut d'Estudis Epacials de Catalunya (CTE‐CRAE/IEEC) Universitat Politècnica de Catalunya Barcelona Spain; ^15^ BioSensе Institute—Research Institute for Information Technologies in Biosystems University of Novi Sad Serbia

**Keywords:** allergy, end‐user needs, fungal spores, high temporal resolution, monitoring, near real‐time, pollen, stakeholders

## Abstract

**Background:**

Information about airborne pollen concentrations is required by a range of end users, particularly from the health sector who use both observations and forecasts to diagnose and treat allergic patients. Manual methods are the standard for such measurements but, despite the range of pollen taxa that can be identified, these techniques suffer from a range of drawbacks. This includes being available at low temporal resolution (usually daily averages) and with a delay (usually 3–9 days from the measurement). Recent technological developments have made possible automatic pollen measurements, which are available at high temporal resolution and in real time, although currently only scattered in a few locations across Europe.

**Materials & Methods:**

To promote the development of an extensive network across Europe and to ensure that this network will respond to end user needs, a stakeholder workshop was organised under the auspices of the EUMETNET AutoPollen Programme. Participants discussed requirements for the groups they represented, ranging from the need for information at various spatial scales, at high temporal resolution, and for targeted services to be developed.

**Results:**

The provision of real‐time information is likely to lead to a notable decrease in the direct and indirect health costs associated with allergy in Europe, currently estimated between €50–150 billion/year.^1^

**Discussion & Conclusion:**

A European measurement network to meet end user requirements would thus more than pay for itself in terms of potential annual savings and provide significant impetus to research across a range of disciplines from climate science and public health to agriculture and environmental management.

## 1 BACKGROUND

Medical practitioners require information about what is present in the atmosphere and how the situation may evolve to better understand and treat their allergic patients. In response to this need, doctors in countries across the globe established measurement sites to monitor airborne pollen concentrations. As the number of allergy sufferers has grown over the past decades^2^, ^3^, ^4^ so have the number of such sites (>500 sites in Europe^5^; 251 sites in Europe^6^) and from the mid‐1980s onwards many of these sites have been organised into regional and national networks. In parallel, fungal spore monitoring was developed for crop protection purposes (e.g.,^7^, ^8^, ^9^) largely at different sites and often carried out by agricultural organisations rather than health‐related ones. In many European countries, both the pollen and fungal spore monitoring networks are maintained through scant and inconsistent funding sources and sometimes run by private organisations, which has significant negative consequences in terms of the temporal continuity of monitoring, heterogeneous spatial coverage, as well as the quality and availability of data for public information and research. Indeed, some networks are at the brink of collapse, with financial or political support lacking and volunteerism having its limits.

Pollen and fungal spore measurements have mainly been carried out using Hirst‐type volumetric samplers.^10^ These impactors collect atmospheric particles on a drum that slowly rotates over the course of several days, usually a week, before it is sent to a lab where it is analysed under the microscope by trained professionals. It is a labour‐intensive and lengthy manual process which usually provides daily‐average data with a delay of anywhere between 3 and 10 days.^11^ Furthermore, these standardised measurements^12^ suffer from a range of errors, including sampling and collection efficiency^13^, ^14^, ^15^, ^16^, ^17^ flowrate estimates^17^ or issues related to the manual counts.^18^, ^19^, ^20^, ^21^ Overall measurement uncertainty is estimated to be at least 30%.^22,23^ Recently, another weakness of this technique became apparent: as the COVID‐19 epidemic shut down countries across the globe, many manual pollen and spore counting sites were unable to continue because personnel were under lockdown. All of this during what was, for the northern hemisphere, the main spring pollen season.

Recent technological developments mean that automatic monitoring of pollen and fungal spores is now a reality. A range of instruments based on different technologies are able to provide data in real‐time (i.e., within minutes or a few hours of the measurement) at high temporal resolution (3 h or less).^47^, ^70^ Such developments are transforming the approach to monitoring not only of pollen and fungal spores, but the full capacity of these instruments is just starting to be explored and a much broader range of particles can likely be identified,^28^ for example, particulate matter levels.^34^

Somewhat similar to what is done for weather forecasts, such high temporal resolution and near real‐time observations can be integrated into numerical models to provide improved spatial forecasts.^35^, ^36^, ^37^, ^38^, ^39^, ^40^, ^41^, ^42^ They also represent crucial information for the qualitative and quantitative retrieval of bioaerosols in the atmospheric column, which has recently been achieved using remote sensing techniques.^43^, ^44^, ^45^, ^46^ In addition, the same technology can potentially also be used for control of indoor air contaminants, for example in hospitals, food processing chains, and so forth.

Together, these advancements are not only revolutionising the information available to health care practitioners and their patients but also provide a range of opportunities beyond the realm of medicine. For example, improved forecasts of certain fungal spore species could reduce the need for pesticide use in the agricultural sector, decreasing both environmental impacts and associated costs (e.g.,^9^). The availability of high temporal‐resolution observations is also opening up a number of research avenues, for example, to better understand the role of pollen and fungal spores in the hydrological cycle (these particles are known to serve as nuclei for cloud and ice droplets) or their role in ecosystem dynamics (see^47^ and references therein;^48^, ^49^).

Within this context, the AutoPollen Programme began in 2018 under the umbrella of the European grouping of national meteorological services, EUMETNET.^50^ This 5‐year programme brings together a wide range of actors from across Europe involved in pollen monitoring, some already with operational automatic monitoring sites (e.g.,^51,52^). AutoPollen aims to establish a prototype of a fully automatic pollen monitoring network, develop standards, foster collaboration across Europe and support wide exchange and use of data. This includes covering the entire information chain, from developing measurement protocols and standards, through designing the optimal network, all the way to ensuring the information produced is effectively communicated to meet the wide range of end‐user needs. To more fully understand these needs, a workshop bringing together players from a range of stakeholder groups was held. This paper aims to describe the needs of these different end‐user groups for real‐time pollen and fungal spore information, as well as to outline the infrastructure and services required to meet these needs.

## 2 METHODOLOGY

Involving end‐users from the outset is a priority for the AutoPollen Programme and, in this respect, representatives from the European level were invited to a Stakeholder Workshop which took place on 3 March 2020 in Brussels, Belgium. This workshop brought together a group of end‐users from various communities, including public health, different domains of research, medical doctors, governmental institutes, meteorological services and patient organisations. The main objectives were to: (1) make end‐users aware of developments and the new possibilities that automatic pollen and spore monitoring provide; (2) establish what information is useful to each end‐user group and how they would like to receive this information, (3) understand what infrastructure is necessary to ensure high‐quality products and services are delivered across Europe; and (4) bring added‐value through the discussions and collaborations between the diverse group of end‐users. Through a range of interactive techniques such as brain‐storming and a ‘world café’, the participants shared their perspectives and experiences. After the workshop, this position paper was drafted to summarise the main conclusions of the workshop together with existing literature and was circulated to all participants for review and final approval.

## 3 USER NEEDS

### 3.1 The health sector

The most important sector and essentially the original ‘raison d'être’ for pollen monitoring networks was to provide information to health practitioners treating allergy sufferers. General practitioners, allergologists, pneumologists and clinical immunologists need to know what pollen and fungal spore taxa are present in the atmosphere to diagnose what their patients are exposed to, prescribe the correct medication and evaluate whether any treatment (antiallergic medication or allergen immunotherapy [AIT]) is effective.^53^ Furthermore, such information has been requested by medical authorities to have clear definition of time intervals for monitoring efficacy in clinical‐development programs of AIT strategies^53^ and clinically justified thresholds for determining these intervals have been harmonised by scientific organisations.^54^ The costs associated with seasonal allergies in Europe are estimated at anywhere between €50–150 billion per year,​^1^, ^55^ so even just a 0.1% decrease as a result of improved treatments would equate to savings of up to €150 million per year. Furthermore, recent research has indicated that the presence of pollen can reduce innate antiviral immunity independent of allergy,^22^ making it even more important to be aware of what is present in the atmosphere.

New automatic monitoring techniques make it possible to provide high temporal resolution information about the quantity of pollen and fungal spores in near real time. These data would provide the opportunity to develop a significantly improved understanding of the relation between symptoms, atmospheric concentrations and exposure levels. Beyond those who are already aware that they suffer from either pollen or fungal spore allergies, there are many who may not even know that they are allergic or to what they are allergic. The provision of real‐time high temporal resolution information will enable this group to better understand their symptoms and the possible relationship with airborne pollen and fungal spore concentrations. Services providing this information will also be required to educate potential users to this extent.

Just as for many air pollutants, recent studies have shown that for certain pollen taxa, there are marked diurnal cycles at the surface^56^ as well as in the atmospheric column^45^ and thus when only daily average values are provided, as is currently done in most places, daily peaks are missed. The relationship between diurnal variations in atmospheric pollen and fungal spore concentrations, exposure (related to patient behaviour) and symptoms has to date never been studied at such high temporal resolution. The provision of high temporal resolution automatic observations would finally allow extensive investigations into this issue, potentially leading to better management and treatment of allergy symptoms. Furthermore, the higher temporal resolution data would allow better understanding of the importance of weather and air pollution levels, both cofactors affecting allergic responses.^57^, ^58^, ^59^, ^60^ Enhanced understanding of such exposure levels will thus significantly improve the possibilities to develop new therapies.^54^ Additionally, several studies have emphasised the occupational and public health risks from bioaerosols emitted from composting facilities, for example, inflammation of the respiratory tract, aspergillosis or allergic and toxic nonallergic asthma.^42^, ^61^ Continuous real‐time monitoring of such bioaerosol in the vicinity of such sources would serve to protect surrounding populations through, for example, the issuance of alerts and warnings.

For the health sector, data are needed at various temporal resolutions (see Table 1). Allergy sufferers may need information a few times a day, at moments when they change their behaviour, for example, in the morning when planning activities. For these end‐users having more measurement sites in space would be more relevant, to ensure that they have information about local conditions. This is also true for medical practitioners and researchers, who in addition also require higher temporal resolution observations to better understand exposure levels and relationships with symptoms.^63^ For their purposes, it is thus important that the observational network is comprised of stations that provide information representative of general population exposure at relatively high temporal resolution (several observations per day). Concomitant exposure to airborne pollen, fungal spores and air pollutants can aggravate allergy symptoms.^58^, ^59^, ^64^, ^65^, ^66^ Therefore complementary observations of other air pollutants, such as ozone or particulate matter, should also be available to fully communicate overall exposure levels^63^ to the public, medical practitioners, and all relevant stakeholders; this is a prerequisite for understanding effects on sensitive individuals.^67^

**TABLE 1:** Summary of currently available pollen and fungal spore observations (in blue) and end‐user needs for four principle stakeholder communities (in yellow)

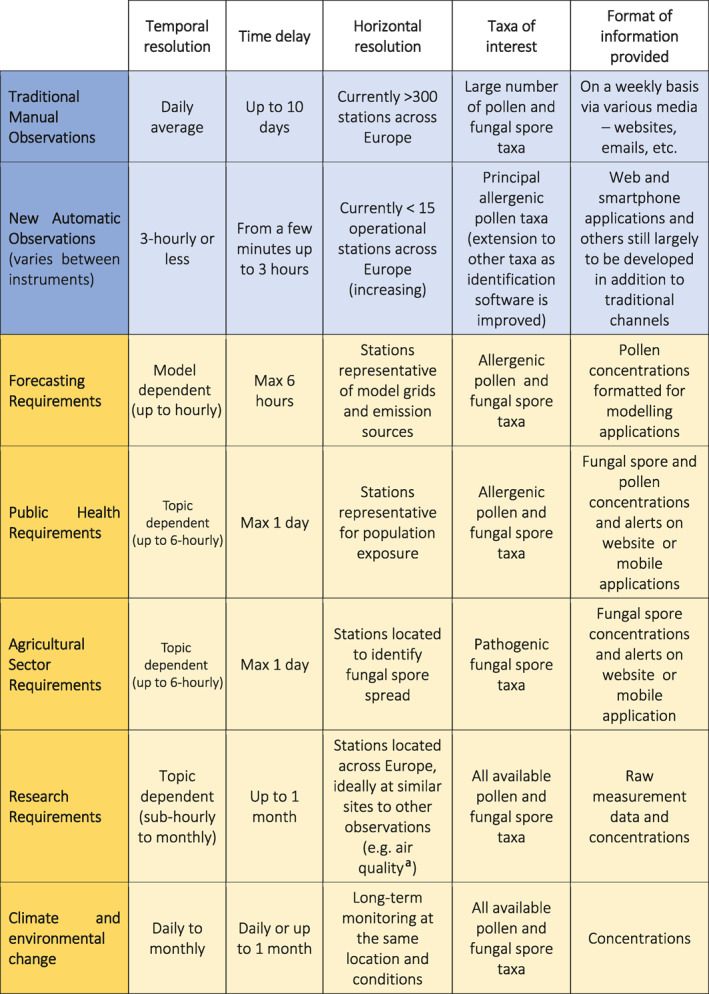

*Note:* Note that for the “Taxa of interest” column, this reflects the current situation in terms of observations (blue rows) and the end‐user needs (yellow rows).

^a^ Air quality stations must be positioned <4 m above ground due to EU‐regulations, whereas pollen monitoring stations should ideally be located at least 10 m above ground.^18^, ^62^

### 3.2 Pollen and fungal spore forecasts

In contrast to observations, forecasts are predictions or projections of the future, even if often they use measurements of current or climatological conditions as a starting point. They are based either on statistical or numerical models, with either method based on observations to a lesser or greater extent. At present, numerical forecast models only use climatologies of relevant pollen and fungal spore seasons, and forecasts are not directly based on current observations since the measurements are usually made available with a delay of 3–9 days. These climatologies are used to provide phenological functions for the particular pollen or fungalspores that are simulated by the forecast models. The phenology functions provide start and end dates, as well as the intensity of the emission process and depend on both present and past meteorological conditions (temperature, relative humidity, precipitation and wind speed). The phenological functions are a key aspect of the emissions schemes used in numerical models. A review of emission schemes for pollen and spores can be found in Vélez‐Pereira^68^, ^69^ while the establishment of phenology functions has been studied in detail recently by Majeed.^70^

Never before has it been possible to integrate pollen and fungal spore observations into forecasts in real‐ or near real time. The availability of such high temporal resolution observations is, therefore, providing an opportunity for a paradigm shift in terms of what is possible. For example, these data can be used to update numerical model fields to current conditions^35^, ^36^, ^37^, ^38^ as is done for meteorological forecasts especially in the context of the European Copernicus programme. The data would need to be provided at temporal resolutions relevant to the respective forecast system used (Table 1). Timely measurements will lead to significantly improved forecasts, since these forecasts will be based on actual conditions, and in turn, that the forecasts can also be updated more regularly, as is done for weather forecasts. This would ensure that punctual events, such as large thunderstorms, could be taken into account in forecasts and allow the emission of alerts in real‐time. This would potentially help reduce the impact of events such as asthma epidemics associated with massive thunderstorms.^57^ Furthermore, these same high temporal resolution measurements are also useful for model validation to assess the accuracy of forecasts compared to actual observations.

Pollen and fungal spore forecasts are, like the initial observations, used by a similar range of end‐users as outlined in this paper. During the workshop, participants indicated that they require forecasts at various timescales, from subdaily through to seasonal. Short‐term forecasts, available each 6 h or at moments in the day when people are likely to change their behaviour (morning/midday/evening), would be highly valuable to allergy sufferers. For example, allergic individuals would be able to better plan their daily activities to avoid periods of peak pollen or fungal spore levels, which are known to show diurnal variability (e.g.,^56^, ^71^). Accurate forecasts available a few days in advance would also allow allergic individuals to appropriately take preventative medication rather than taking it once symptoms appear. Likewise, accurate forecasts on this timescale would be useful for advising allergic individuals when travelling across Europe. This raises challenges regarding the thresholds to which different populations are sensitive^72^ and how to communicate this information; however, further research on this topic is required. Finally, seasonal forecasts, even information as simple as providing probabilities that the season is expected to be more or less intense than normal, as well as expected start and end dates, would be very valuable to medical practitioners and their patients. Such information would allow patients and their doctors to be better prepared, with patients being able to take allergy medication in a more targeted manner and their doctors to improve how immunotherapies are applied. More precise forecasts of season start and end dates would also be beneficial to the pharmaceutical industry and pharmacies to improve the supply chain management of allergy medication. Such long‐term forecasts would also allow advance planning of primary environmental protection measures, for example, in the control of ragweed.

For forecast models, it is particularly important that observational sites are located in source regions so that the simulated emissions can be updated using near real‐time observations (see Table 1). This is in contrast to the needs of the health sector, which require stations rather to be representative of population exposure and thus closer or even in areas where people live. The influence of emission sources, for both pollen and fungal spores, is highly localised, so it is realistic to imagine a network that is similar to that of the current pollen monitoring network (see^5^). This would eventually entail 200–300 automatic stations strategically spread across Europe to cover the main bioclimatic zones and emission source regions. In addition to just the airborne concentration values, it is particularly important for forecast models that each measurement is reported with an uncertainty value since this information is vital to be able to properly assimilate observations and to evaluate forecast accuracy.

### 3.3 Agriculture and forestry

Fungal spores are common crop pathogens that cause disease and significant economic losses every year. Epidemics of such diseases are known to be associated with wind.^73^ In the food storage and processing chain, these species also cause important damages and losses. Estimates from the early 2000s showed that of the annual worldwide food production between 31% and 42% (approximately US$ 500 billion) was lost as a result of diseases, weeds and insects, while an additional 6%–20% (approximately US$ 120 billion) was lost postharvest because of insects and fungal and bacterial rots.^74^

Monitoring the spread of fungal spores in real time can help reduce infection rates by allowing early preventive measures to be taken (e.g.,^7^, ^8^) as well as targeted rather than widespread use of fungicides. Overall, this contributes to a reduction in the amount of toxic products used, thus also decreasing environmental impacts and associated costs (e.g.,^9^, ^75^ collection efficiency). In addition, early detection of the occurrence and spread of airborne plant pathogens may help prevent large‐scale epidemics of diseases such as ash dieback.^76^ Despite the obvious benefits of having aerobiological data to manage such plant diseases, only few disease control systems utilise airborne fungal spore monitoring, for example, for the control of *Venturia inaequalis* or *Phytophthora infestans*.^77^ To be useful for plant disease management, aerobiological measurements need to be timely (alerting about the presence of pathogens in real time), specific (able to identify particular pathogens of relevance) and sensitive (able to detect small quantities of spores before the disease can be visually observed).^78^ Advancements in flow cytometry‐based methods for bioaerosol monitoring provide the possibility to meet all three criteria since they permit specific analysis of single particles and high sampling rates. Depending on the crop and disease, local‐scale monitoring may be required for precise disease management^73^ and emission characterisation, but coupling measurements with atmospheric models is expected to provide more information about spatial variations in exposure risk and should serve as an additional tool to control and manage agricultural pathogens.

### 3.4 Research

The high temporal resolution of automatic pollen and fungal spore measurements provides the possibility for a quantum leap in terms of the research that is possible. Domains as diverse as epidemiology, atmospheric physics and agronomy, amongst many others, would significantly benefit from such information. The door is also open to a wide range of other fields where the application of such data has not yet been explored.

High temporal resolution observations provide the possibility to better understand the subdaily variability of pollen and fungal spore concentrations, such as how they are dependent on meteorological factors or how their emissions vary over the course of the day. This is intimately related to the establishment of phenological functions of each pollen/spore taxa, in turn crucial information for the forecast models mentioned earlier. In terms of health research, such data would be useful to study how the behaviour of allergic individuals varies in time compared to atmospheric pollen or fungal spore loading, as well as to better understand the relationship between symptoms and exposure levels, sensitisation rates and the complex biological mechanisms behind allergies. Other research questions related to new therapies as well as assessing the rate of medication consumption and self‐management in response to the provision of near real‐time information and forecasts would also benefit from such measurements. These studies would in turn be useful to establish better public health practices, to communicate more accurate risk levels and to make recommendations to the general public to further reduce exposure levels.

The availability of high temporal resolution observations and better spatial coverage will also allow significant progress to be made in several other fields, including atmospheric sciences, ecosystem research, understanding the role of pollen and fungal spore particles in the hydrological cycle as well as studies of biogeography and biodiversity. Pollen/spores are present all over the globe and, as any atmospheric aerosol, they scatter and absorb radiation entering the Earth‐atmosphere system. For example, in the region of Barcelona, in strong pollen events, pollen can represent up to 30% of the total optical depth.^45^ Since present remote sensing technology cannot be used to identify pollen from other aerosols, let alone individual pollen taxa, high temporal resolution surface observations are the only way to link surface and atmospheric column observations.

Researchers need high temporal resolution data (see Table 1), although not necessarily in real‐time, except for specific studies where this may be useful. Importantly, not only are the measurements themselves relevant, but as for forecasts, the uncertainty associated with each observation point is also important. Likewise, multifactorial information is important, that is the provision of meteorological or air quality observations from the same or close‐by sites.

### 3.5 Climate and environmental change

In the context of climate change, pollen and fungal spore seasons are expected to become longer,^79^, ^80^, ^81^ with higher allergen levels (even if airborne concentrations remain similar),^82^ and with faster development of certain fungal species in warmer temperatures. Increased CO_2_ concentrations also enable many plants to produce more pollen^83^ and increases the allergenicity of pollen.^84^ Such changes will have obvious impacts on allergy, human health and agriculture, to name but a few. For example, projections for Europe of the rate of Ragweed sensitisation show that climate change is likely to double the level from 33 million people (1986–2005) to 77 million by 2041–2060.^58^ The availability of high temporal resolution observations as well as better spatial coverage of the network will help better understand the dynamics of invasive species, such as ragweed, by providing information that can be used to understand and identify sources (local or transported) using techniques such as back‐trajectories. In this sense, the retrieval of pollen information in the atmospheric column using advanced lidar techniques,^64^, ^72^ together with the coordination with extended atmospheric observational networks, such as ACTRIS (Aerosol, Cloud and Trace Gases Research Infrastructure, www.actris.eu), will boost research in this domain.

Understanding any current or future climate change requires a baseline for comparison and it is thus also important that historical observations, even if manual, are made freely available from across the European continent. This is presently not the case for many observations sites, and there is a risk that these data, particularly the oldest parts of records, are lost. While not the focus of this paper, it is important to point out the significance of these data, together with new automatic observations carried out in the long‐term (see Table 1), particularly for climate change research and forecast model validation purposes.

## 4 RESPONDING TO END‐USER NEEDS—THE IDEAL PAN‐EUROPEAN POLLEN MONITORING NETWORK

**FIGURE 1:**
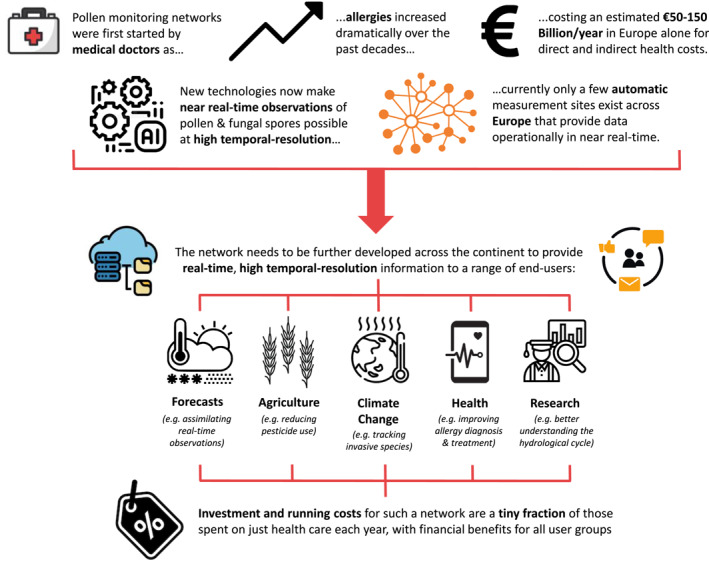
Schematic of current and developing automatic pollen and fungal spore monitoring across Europe as well as example end uses

To meet the end‐user needs outlined above, a comprehensive strategy and integrated infrastructure needs to be put in place (see Figure 1). Existent automatic monitoring networks^51^, ^52^ as well as those in the development or planning stages are currently collaborating together through the EUMETNET AutoPollen Programme.^50^ While this programme is coordinating the nascent network of stations, this will need to be reinforced and further extended to form a ‘backbone’ network across all of Europe. This network will ultimately require at least 200–300 representative sites strategically located across Europe covering the main bioclimatic zones and providing data that can be used for forecasts at the continental scale. This ‘backbone’ network will need to be complemented by more dense regional or local networks, which in turn can provide more detailed information, for example, in more populated areas where airborne pollen and fungal spore concentrations have been shown to be heterogeneous in space.^85^, ^86^ While certain regions can afford the initial investment that automatic monitoring entails, others are not yet able to do so. In time, such gaps will need to be filled to ensure that accurate information, both high temporal resolution observations and improved numerical forecasts, can be provided to the entire European population.

The airborne concentration data that these networks produce need to be made available through a common database open for research, numerical forecasts and public use. This will lead to the development of a range of targeted products and services for all end‐user groups, whether this be innovative smartphone applications or dedicated informational websites. The whole value chain thus should to be considered (e.g.,^87^)—from the initial measurement all the way through its interpretation, model forecasts and to the final services provided to end‐users.

While not directly an end‐user need, pollen and fungal spore monitoring currently suffers from the challenge of not being a regulatory requirement in most countries, as is the case for most air pollutants for which there are strict monitoring standards and statutes. One of the main reasons for this situation is that pollen and spores are considered ‘natural' and therefore controlling their production is thought of as impossible. However, in certain areas this is no longer the case. For example, particularly in densely populated areas, vegetation is often not at all natural; it is either planted (e.g. birch or cypress) or accidentally introduced by anthropogenic activities (ragweed being a typical example of this). Thus, the possibility to control and regulate such vegetation and the allergenic pollen it produces is essential. Finally, the continuous increase in the number of allergy sufferers means there is a growing need for the allergy‐causing substances to be monitored. The development of regulations to make such monitoring mandatory across Europe would significantly aid the establishment and maintenance of an observational network.

## 5 CONCLUSIONS—THE FUTURE IS NOW

Preparedness of health systems can be strengthened by improving the preparedness of health professionals, which includes providing them with better information and services upon which they can make decisions. This can naturally also be extended to the public who also need to be informed about observed and projected changes in environmental exposure, such as shifting pollen seasons or extension of the geographical distribution of allergenic plant taxa.

A unique window of opportunity exists to establish an automatic pollen and fungal spore monitoring network across Europe in a standardised, collaborative and coherent fashion. It is essential that national and regional efforts to establish observation sites are coordinated, that common measurement protocols are followed and that the real‐time information is made freely available in ways that meet end‐user's needs. The EUMETNET AutoPollen Programme aims to achieve this through establishing a prototype network across Europe that provides end‐to‐end solutions for a range of stakeholders. This more operationally oriented programme is being run in parallel and in synergy with ongoing research and development projects, for example, within the Copernicus Atmospheric Monitoring Service or the Cost Action ADOPT (new approaches in detection of pathogens and aeroallergens). Such collaboration is essential to ensure that the potential of current measurement capabilities is fully exploited, for example by extending the number of pollen and fungal spore taxa that can be identified, and that the newest techniques and methods are taken up operationally. This could include complementary measurements of aeroallergens, whose concentrations may vary in each pollen grain or fungal spore 20, or for measurements of indoor biological air quality, where there are a wide range of potential uses to be explored, such as in hospitals or food processing chains.

The long‐term sustainability of the European network is vital and ideally should be ensured through engagement from government organisations with stable funding sources and under long‐term mandates. Given that the direct and indirect health costs associated with allergy and asthma are estimated to be between €50–150 billion per year in Europe,^1^, ^88^ even just a 0.1% decrease in these costs would equate to cost savings of up to €150 million per year. In terms of cost‐benefit ratios, the network would thus more than pay for itself. Furthermore, the measurements from the network could be used in a range of other domains such as agriculture, where considerable cost‐savings are also possible (e.g.,^9^) or to further a wide spectrum of research in areas such as climate change, public health and environmental management.

## CONFLICT OF INTERESTS

The authors declare that there are no conflict of interests.

## AUTHOR CONTRIBUTIONS

Fiona Tummon and Bernard Clot wrote the manuscript. All other coauthors contributed to the further development of the text. All authors read and approved the final manuscript.
